# Centrality nearest-neighbor projected-distance regression (C-NPDR) feature selection for correlation-based predictors with application to resting-state fMRI study of major depressive disorder

**DOI:** 10.1371/journal.pone.0319346

**Published:** 2025-03-06

**Authors:** Elizabeth Kresock, Bryan Dawkins, Henry Luttbeg, Yijie (Jamie) Li, Rayus Kuplicki, B. A. McKinney

**Affiliations:** 1 Tandy School of Computer Science, The University of Tulsa, Tulsa, Oklahoma, United States of America; 2 SomaLogic, Inc., Boulder, Colorado United States of America; 3 Department of Mathematics, The University of Tulsa, Tulsa, Oklahoma, United States of America; 4 Laureate Institute for Brain Research, Tulsa, Oklahoma, United States of America; University of Pittsburgh, UNITED STATES OF AMERICA

## Abstract

**Background:**

Nearest-neighbor projected-distance regression (NPDR) is a metric-based machine learning feature selection algorithm that uses distances between samples and projected differences between variables to identify variables or features that may interact to affect the prediction of complex outcomes. Typical tabular bioinformatics data consist of separate variables of interest, such as genes or proteins. In contrast, resting-state functional MRI (rs-fMRI) data are composed of time-series for brain regions of interest (ROIs) for each subject, and these within-brain time-series are typically transformed into correlations between pairs of ROIs. These pairs of variables of interest can then be used as inputs for feature selection or other machine learning methods. Straightforward feature selection would return the most significant pairs of ROIs; however, it would also be beneficial to know the importance of individual ROIs.

**Results:**

We extend NPDR to compute the importance of individual ROIs from correlation-based features. We introduce correlation-difference and centrality-based versions of NPDR. Centrality-based NPDR can be coupled with any centrality method and can be coupled with importance scores other than NPDR, such as random forest importance scores. We develop a new simulation method using random network theory to generate artificial correlation data predictors with variations in correlations that affect class prediction.

**Conclusions:**

We compared feature selection methods based on detection of functional simulated ROIs, and we applied the new centrality NPDR approach to a resting-state fMRI study of major depressive disorder (MDD) participants and healthy controls. We determined that the areas of the brain that have the strongest network effect on MDD include the middle temporal gyrus, the inferior temporal gyrus, and the dorsal entorhinal cortex. The resulting feature selection and simulation approaches can be applied to other domains that use correlation-based features.

## Background

Resting-state functional MRI (rs-fMRI) measures the blood oxygen level-dependent (BOLD) signal in regions of interest (ROIs) throughout the entire brain of a subject while at rest (i.e., not during a cognitive task) [1–4]. The BOLD signal for each ROI is a time series whose low-frequency fluctuations are correlated with other ROIs. These correlations can be used to represent the functional connectivity (FC) between ROIs in a weighted brain network [5–9]. Differential changes, or rewiring of FC, between subjects with mood disorders and healthy controls may reveal neural mechanisms of disease. Feature selection and machine learning methods that use FC measures as predictors have potential as fMRI-based biomarkers and for disease classification [10]. A wide variety of machine learning (ML) algorithms, including support vector machines (SVMs), XGBoost, random forests, and deep learning have been used widely for rs-fMRI data to detect and better understand the mechanisms of mood disorders, including major depressive disorder (MDD) [11]. Various biomarkers and measures of FC between brain regions have been used as fMRI-based ML pred [1–4] ictors, including correlation, mutual information, amplitude of low frequency fluctuation (ALFF) and regional homogeneity (ReHo). ML classification of MDD with rs-fMRI has been promising, but using ML for diagnosis is likely premature [11]. Feature selection, which is the focus of the current study, while not directly diagnostic, may provide valuable insights into the biological mechanisms of MDD.

As the input variables for feature selection, we use preexisting ROIs based on brain atlases constructed by experts using anatomical and functional information. Feature engineering methods, such as independent component analysis (ICA) to define brain networks and ROIs [12], may capture additional variation but may be more difficult to interpret. Similarly, multivoxel pattern analysis (MVPA) [13] uses an SVM to build classifiers without assumptions about the organization of the brain, but the distributed collection of voxel associations may also be difficult to interpret and difficult to generalize between datasets.

We use Pearson’s correlation coefficients across the full time series between pairs of brain regions as the predictors for feature selection. Correlation is a convenient way to quantify FC and represent temporal synchrony of ROI activity. A common way to identify important ROIs from correlation FC is to perform a seed-based analysis, where the global correlation between a given seed region and all other brain regions is computed [14]; then, this centrality quantity can be tested for all ROIs for association with an outcome such as MDD. In the present study, we use centrality in a different way with our nearest-neighbor projected distance regression (NPDR) approach [15]. We apply NPDR to correlation-based predictors and then apply network theory to determine the cumulative effect of the differential correlations for each ROI. We also integrate this centrality approach with random forest (rf) in a new centrality rf (c-rf).

This machine learning feature selection study for correlation-based features is outlined as follows. We review the relevant ideas behind the penalized and nonpenalized NPDR feature selection method and describe the new distance metrics for compatibility with correlation-based features. We emphasize that our goal is to understand not only the importance of pairs of variables for predicting outcomes but also the importance of individual variables in the network. Next we describe a new simulation strategy for correlation-based data for classification tasks, and then we describe a real correlation-based dataset in the form of a previous rs-fMRI study of MDD. We compare the performance of the feature selection methods using the new simulation tool and the real rs-fMRI study of MDD, and we discuss the implications of some of the brain ROIs found to be associated with MDD.

## Methods

**Fig 1:**
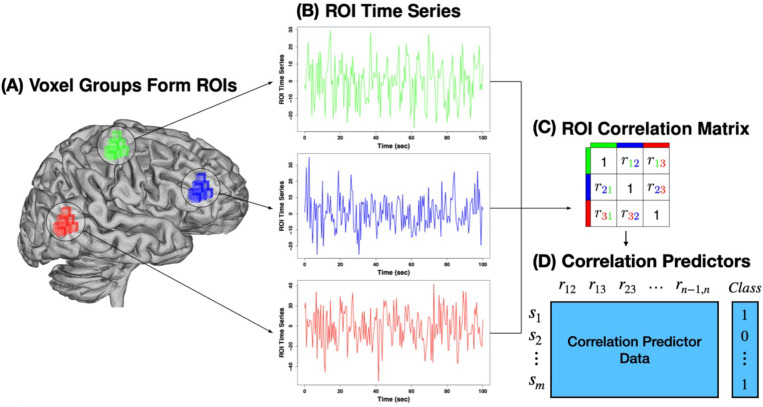
Illustration of resting-state fMRI data used for machine learning feature selection. Regions of interest (ROIs) are composed of groups of voxels within the brain. Three ROIs (A) are used for illustration (green, blue, and red cubes/voxels), but the number of ROIs is typically on the order of 200. Each voxel has an associated time series, which is averaged within ROIs to create the green, red and blue time series (B). From these time series, pairwise ROI correlations are calculated and stored in a matrix for each subject (C). The upper triangle of each subject’s correlation matrix can be stretched into a sample vector, s_i_, to form rows of a dataset (D), where the predictors (columns) are ROI-ROI correlations.

Our goal is to identify important variables or pairs of variables that are important for predicting a given outcome variable. However, we assume that predictor information is only given in the form of pairwise correlation. The type of data we have in mind is correlation between brain regions in rs-fMRI studies. For a subject (Fig 1A), we average the voxel time series in each ROI of on a brain atlas (Fig 1B) and then compute the correlation between all pairs of n ROI time series (Fig 1C). For each subject, the upper triangle of the correlation matrices is stretched to create datasets, where the n(n-1)/2 predictor variables are pairwise correlations between ROIs (Fig 1D). Standard feature selection methods can then be used for these data to determine the importance of ROI pairs. In addition to individual ROIs, the current feature selection approach aims to disentangle the many important pairs to identify important individual ROIs. Nearest-neighbor projected-distance regression (NPDR) is a machine learning feature selection algorithm that is able to detect statistical interactions using nearest neighbors in a high-dimensional space [15]. Before describing the centrality-based and correlation-distance extensions of NPDR, we review some of the relevant aspects of NPDR for ranking the importance of variables for predicting a class variable. NPDR minimizes the contrastive loss function for pairs of samples $\left(i,j\right)$ . The contrastive loss $\delta_{ij}\left(y\right)$ is an indicator of whether sample pair $\left(i,j\right)$ is in the same class or a different class based on class variable *y*. The contrastive loss can be penalized by LASSO or Ridge, or it can be unpenalized and P-values can be computed. Rather than using the predictor/attribute values directly in the regression, NPDR uses the difference ${\vec{d}}_{ij}\left(X\right)$ (or projected distance onto the attribute matrix X) between subjects $\left(i,j\right)$. The vector denotes the projected distance for all attributes in the set X. In the current application, the attributes are Pearson correlations between pairs of ROIs. For centrality NPDR (c-NPDR), the projected distance or diff, $d_{ij}\left(p\right)$, is the absolute difference between subjects $\left(i,j\right)$ for one correlation attribute *p* (correlation between a pair of ROIs):.


$$d_{ij}\left(p\right)=\left|A_{p}^{\left(i\right)}-A_{p}^{\left(j\right)}\right|$$ (1)

where $A_{p}^{\left(i\right)}$ is the correlation for subject *i* between a pair of ROIs, represented by *p*. Thus, if there are *n* ROIs, the NPDR design matrix consists of n(n-1)/2 attribute columns; one for each pair of ROIs. The NPDR-selected ROI pairs can then be used in any number of centrality algorithms to rank the importance of individual ROIs. For comparison, we use the following centralities: degree, betweenness, eigenvector and Integrated Value of Influence (IVI) [16].

The other NPDR-based method (correlation-diff-NPDR) for ranking the importance of ROIs from ROI-pair correlation data uses a more complex projected distance, $d_{ij}^{CD}$, but directly gives importance of individual ROIs without centrality calculations [17]. The correlation-diff (CD) or correlation projected distance for ROI *r* is given by


$$d_{ij}^{CD}\left(r\right)=\underset{k\neq r}{\sum }\left|A_{rk}^{\left(i\right)}-A_{rk}^{\left(j\right)}\right|$$ (2)

where $A_{rk}^{\left(i\right)}$ is the correlation between ROIs *r* and *k* for subject *i*. Thus, the correlation-diff for ROI *r* is the absolute sum of the differences between *r* and all other ROIs. If there are *n* ROIs, the NPDR design matrix for Eq. (2) will have *n* columns as opposed to *n(n-1)/2* for Eq. (1). Thus, NPDR with Eq. (2) yields importance scores for ROIs, while NPDR with Eq. (1) yields the importance of ROI pairs. In both cases (Eqs. 1 and 2), NPDR importance can be computed in terms of individual P-values, which we adjust for false discoveries, or in a multivariate model with LASSO or Ridge regression.

**Fig 2:**
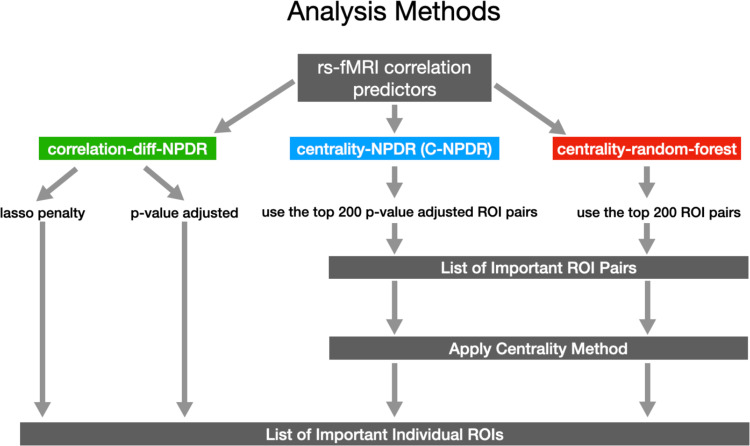
Analysis methods for rs-fMRI data with correlation-based features and a class variable. On the left, correlation-diff-NPDR (Eq. 2) can directly rank the importance of ROIs using P-values or penalized regression coefficients. On the right, centrality-NPDR (C-NPDR, Eq. 1) and centrality random forest (c-rf) rank the importance of pairs of ROIs, and then the centralities of the resulting ROI-ROI networks are used to rank the importance of individual ROIs.

To threshold the results of correlation-diff-NPDR and cNPDR, we use regularization and FDR adjusted P-values. We use the LASSO penalty, also known as the L1 penalty, which is a regularization technique used in regression models to prevent overfitting and to enhance the model’s prediction accuracy and interpretability. For non-penalized methods, we use a P-value cutoff adjusted for multiple testing, where ROI pairs that had an adjusted P-value >  0.05 are removed from the network. For random forest importance, we use a cutoff of the top 200 pairs of ROIs (Fig 2) because there is not a clear statistical threshold.

Correlation-diff-NPDR directly yields a list of significant individual ROIs. However, centrality methods need an additional step to map pair importance to individual importance. The c-NPDR and c-rf methods yield lists of important pairs of ROIs, so we apply centralities to the resulting edge lists to obtain a list of important individual ROIs (Fig 2). The significant pairs of ROIs are graphed as a network, where the nodes are ROIs and edges are defined when the ROI pairs have a correlation that affects the outcome variable (e.g., MDD). This interaction network is a way to visualize the importance of MDD nodes based on their connections and visualize local structure. We quantify the importance of individual ROIs using common centralities, degree, eigenvector, betweenness, and IVI [16]. IVI combines multiple centrality measures.

We also implement a centrality random forest (c-rf), which applies the centrality approach to random forest importance of correlation pairs, and we compare c-rf with c-NPDR. In other words, we use the correlation predictor data (Fig 1d) to compute random forest permutation importance with 5000 trees, filter the correlation pairs to the top 200 to create a network, and then compute ROI centralities.

### Simulation method and real data

#### Simulation approach.

**Fig 3:**
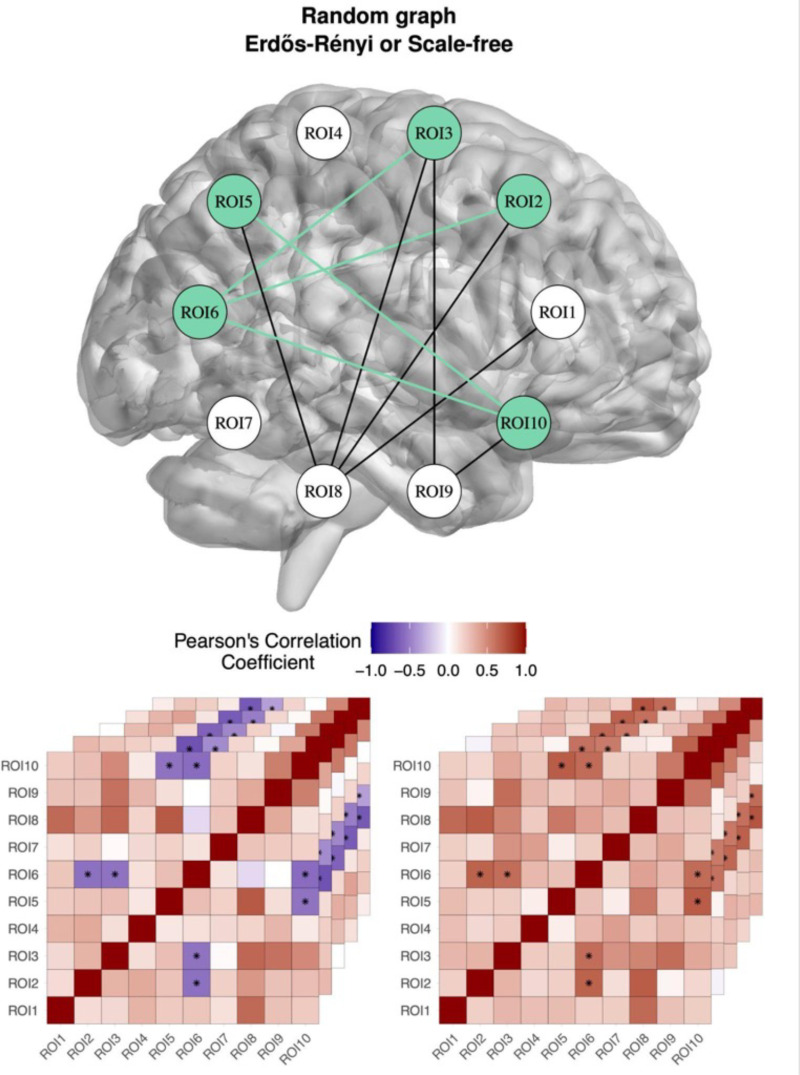
Simulation method for correlation-based features and case-control outcome. A random network is generated (Erdos-Renyi in the example) between the number of regions of interest or ROIs (10 circles in the brain). For each sample, random correlations are mapped to connected regions of the network, and lower random correlations are mapped to unconnected regions. Pairs of regions are selected to be functional (associated with the outcome variable) and are indicated by green edges in the brain network and black dots in the heatmaps. Each heatmap represents a different sample. For the cases (left heatmaps), the selected functional pairs are perturbed to have a higher correlation, and for the controls (right heatmaps), the functional pairs are perturbed to a lower correlation. The final heatmaps represent case‒control datasets with correlation-based features containing noise and functional ROI pairs.

We develop a random network approach to simulate correlation-based features, a fraction of which are functional or associated with case–control status (Fig 3). The application we have in mind is correlation between brain ROIs in resting-state fMRI studies, where correlation is calculated from the BOLD signal time-series. We do not simulate the time series but rather directly simulate the correlations and their differences between groups. Features or predictors are correlations between pairs of ROIs rather than ROIs themselves. We note that these simulations and feature selection methods can also be applied to other types of correlation-based data in other research domains.

The simulation includes parameters that control the number of ROIs, the number of cases and controls, the number of functional ROIs (i.e., those associated with the outcome), the effect size and the type of underlying random network for the brain. In the current study, we specify an Erdos-Renyi network, but the simulation can generate any network from the igraph library. Furthermore, the simulation software allows a user to input their own network; for example, based on real correlation data. Initial correlation matrices are generated for each sample based on the input network, where connected ROIs have higher random correlations than unconnected ROIs.

Functional nodes are chosen from the largest connected component (i.e., the group of nodes such that there is a path between any pair of nodes in the group). Edges between the functional nodes (green edges in Fig 3) are subsequently used to create differential correlations between the cases and controls (black dots in Fig 3 heatmaps). We use a parameter called “multiway” that controls how many edges we randomly select to generate differential correlations. For example, a multiway of 2 will use only a subset of the possible edges between functional nodes (a subset of possible edges will be green). If we set multiway to the maximum, then all possible edges between functional nodes will have differential correlations (green). We use multiway = 5 in this application. We generate replicate simulations to compare feature selection methods based on the ability to detect simulated ground truth functional ROIs. We use the F1 score to test whether the top ROI features selected by a method overlap with the top functional features.

#### Real data.

We compare feature selection methods on data from the Tulsa 1000 (T1000), a longitudinal study at the Laureate Institute for Brain Research following 1000 individuals, including healthy individuals and those with mood and other disorders [18]. We use rs-fMRI time series for 188 MDD subjects and 47 healthy controls (HCs) from T1000 (163 females and 72 males). Cardiac- and respiration-induced noise reduction RETROICOR preprocessing were applied to the time series along with despiking and regressing out low-frequency, 12-motion parameters, local white matter average signal (ANATICOR). Subjects with RMS motion larger than 0.2 were excluded from the analysis.

We use the Automated Anatomical Labelling (AAL) Atlas with 87 ROIs and the Brainnetome Atlas with 246 ROIs to define consistent and interpretable mappings for selected features [19,20]. The Brainnetome Atlas parcellates the brain based on structural and connectivity features. Neuroimaging data, particularly rs-fMRI and diffusion tensor imaging (DTI) data, are used to reveal both functional and structural connectivity patterns in the brain. For each atlas, we detrended the signals and averaged the time series for the voxels within an atlas ROI.

## Results

We simulate 50 replicate datasets each with 100 cases, 100 controls, and 100 ROIs. We select 10 functional ROIs, but their effects are detected through their correlations with each other in correlation-predictor datasets. The underlying correlation networks are based on Erdos-Renyi random networks with connection probability p = 0.1. We use a medium effect size of 0.5 Cohen’s d.

**Fig 4:**
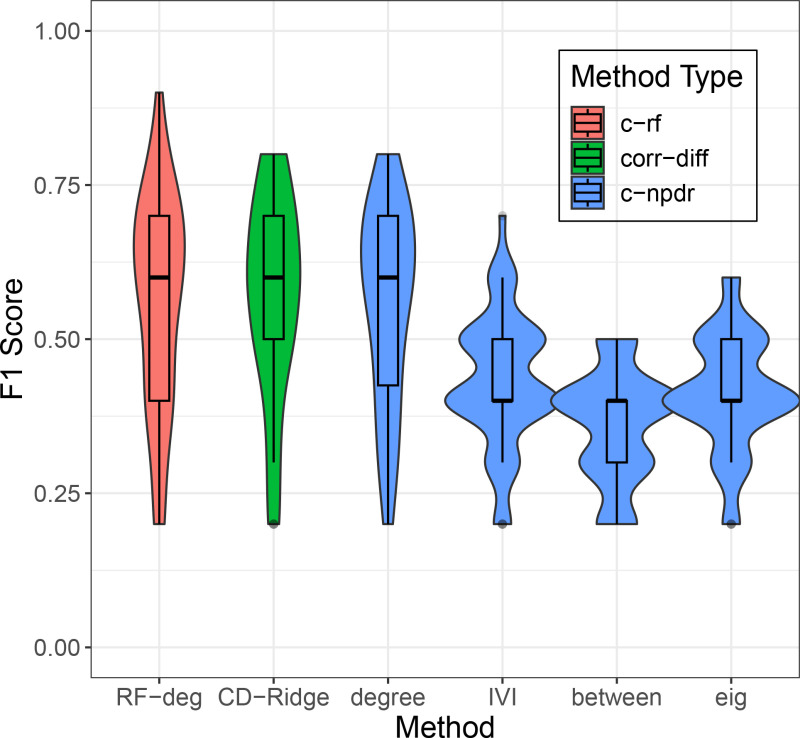
Comparison of feature selection methods for identifying simulated top 10 functional ROIs (out of 100) associated with the class variable. The F1 score is used to quantify whether the top 10 ROIs ranked by a given method are enriched for the top 10 simulated functional ROIs. Each violin plot represents 50 replicate simulations created using random network theory (Fig 3). Colors (indicated in legend) represent three main types of ROI feature importance: degree centrality with random forest (c-rf, red), NPDR using the Eq. 2 metric and Ridge regression (corr-diff, green), and NPDR using the Eq. 1 metric followed by centrality (c-ndpr, blue). For the c-npdr methods (blue), we use four centralities: c = degree, IVI, betweenness and eigenvector. The corr-diff NPDR method (green) directly ranks ROIs as opposed to using centrality from ROI-pair scores, and its performance is similar to centrality random forest (red c-rf, c = degree) and centrality npdr with degree (blue c-npdr, c = degree). The centrality-NPDR methods that do not use degree (three blue plots on the right) perform significantly worse (P < 0.05) than corr-diff NPDR, degree c-rf, and degree NPDR (three plots on the left).

We apply six feature selection methods to the replicate simulations (Fig 4) and compare them based on their average ability to detect the 10 functional ROIs. Centrality-random forest (c-rf) with degree centrality (red, Fig 4) has a similar mean F1 score to correlation-diff NPDR (corr-diff) using Ridge regression (green CD Ridge, Fig 4), and they are both similar to centrality-NPDR (c-npdr) with degree centrality (left blue). The npdr-based methods, namely, corr-diff and c-npdr with degree, exhibit slightly less variation than does the c-rf method. The F1 scores for centrality-based NPDR methods (all blue, Fig 4) depend on the centrality method used. C-NPDR works best with degree, whereas IVI, betweenness, and eigenvector centralities are noticeably worse. The close similarity between c-npdr (Eq. 1) with degree and npdr with corr-diff (Eq. 2) suggests that the corr-diff metric (Eq. 2) is mathematically related to degree centrality.

We apply four feature selection methods to the real rs-fMRI data to compare the selected important ROIs for MDD (Table 1). Because we do not have ground truth true positive ROIs for MDD, we compare the properties and selected ROIs between methods. The correlation-diff NPDR model with LASSO selects the fewest features because it has a tendency to eliminate correlated features (first column, Table 1). The other correlation-diff NPDR analysis (second column, Table 1) uses an FDR adjusted P-value cutoff rather than LASSO, which results in more selected features in part due to the inclusion of more correlated features. The centrality methods that use degree – NPDR (column 3) and random forest (column four) – use a manual threshold because degree centrality does not have a statistical threshold. Although the focus of this study is feature selection, the random forest out-of-bag classification accuracy using all correlation features is 79.1%.

**Table 1:** Important brain regions of interest (ROIs) for major depressive disorder (MDD) according to different feature selection methods.

	correlation-diff NPDR LASSO	correlation-diff NPDR p-value	centrality NPDR p-value, degree	centrality random-forest, degree
Top ROIs Brainnetome Atlas	MTG_R_4_2	MTG_R_4_2	ITG_R_7_4	PhG_L_6_4
	STG_L_6_1	STG_L_6_1	PhG_L_6_4	STG_L_6_1
	ITG_L_7_3	ITG_L_7_4	MTG_R_4_2	MTG_L_4_2
	ITG_L_7_4	ITG_L_7_3	ITG_L_7_3	FuG_L_3_1
	PhG_L_6_4	ITG_R_7_4	ITG_L_7_4	Tha_R_8_4
	MTG_R_4_3	PhG_L_6_4	STG_L_6_1	PhG_R_6_4
		MTG_R_4_3	OrG_L_6_4	PhG_R_6_5
		PCun_L_4_2	PhG_R_6_4	CG_L_7_2
		MTG_L_4_2	PCL_R_2_1	ITG_L_7_3
		IFG_L_6_2	FuG_L_3_1	OrG_R_6_1
		PCL_L_2_1	ITG_L_7_2	LOcC_L_4_2
		PrG_L_6_4	PCun_L_4_2	OrG_L_6_4
		PCL_R_2_1	PCun_R_4_2	SPL_L_5_4
		PCL_L_2_2	STG_R_6_6	ITG_L_7_4
			MTG_L_4_2	PoG_L_4_3
			MTG_R_4_3	OrG_R_6_4
			INS_R_6_4	pSTS_L_2_1
			IFG_L_6_2	LOcC_L_4_1
			PCun_R_4_4	PCun_L_4_4
Top ROIs AAL	Dorsal_DMN_03	Dorsal_DMN_03	Dorsal_DMN_03	Anterior_Salience_01
	Ventral_DMN_10	Ventral_DMN_07	Ventral_DMN_07	Anterior_Salience_02
	Ventral_DMN_07	Right_ECN_04	Right_ECN_04	Anterior_Salience_03
	Right_ECN_04		Ventral_DMN_05	Anterior_Salience_04
			Posterior_Salience_12	Anterior_Salience_05
			Visiospatial_01	Anterior_Salience_06
			Ventral_DMN_01	Anterior_Salience_07
			Ventral_DMN_06	Auditory_01
			Visiospatial_04	Auditory_02
			Ventral_DMN_10	Auditory_03
			Right_ECN_02	Basal_Ganglia_01
			Left_ECN_01	Basal_Ganglia_02
			Visiospatial_08	Basal_Ganglia_03
			Basal_Ganglia_01	Basal_Ganglia_04
			Sensorimotor_05	Basal_Ganglia_05
			Posterior_Salience_04	Dorsal_DMN_01
			Ventral_DMN_04	Dorsal_DMN_02
			Language_05	Dorsal_DMN_03
			Left_ECN_05	Dorsal_DMN_04

**Fig 5:**
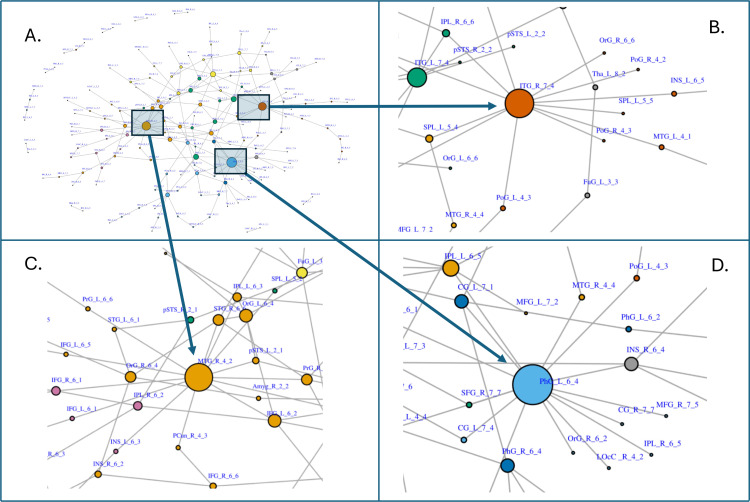
NPDR network of brain regions of interest (ROIs) for major depressive disorder (MDD) association using the Brainnetome (BNA) atlas. The nodes are sized by degree and colored according to Louvain network clustering. Three areas of the overall network (A) are highlighted around the three most important ROIs for MDD according to NPDR (see top of Table 1, column 1). These main ROIs are **(B)** ITG (inferior temporal gyrus), **(C)** MTG (middle temporal gyrus), and **(D)** PhG (parahippocampal gyrus). They are part of separate Louvain clusters (node colors).

For the Brainnetome atlas (top, Table 1), we select the most parsimonious list of ROIs according to NPDR correlation-diff LASSO (column 1), and these features are included in the longer lists of NPDR methods (columns 2 and 3). The random forest method has a slightly different set of selected ROIs because it is not distance based and tends to find more main effects than interactions compared to NPDR methods. We highlight the selected ROIs involving the MTG (middle temporal gyrus) because it is the top ROI found by NPDR correlation-diff (Table 1). MTG has been associated with MDD in previous studies [21,22]. The highest scoring ROIs for the BNA atlas (top, Table 1) correspond to hubs in the NPDR network of ROI pair scores (Fig 5A). The three hubs are inferior temporal gyrus (ITG, Fig 5B), MTG (Fig 5C), and parahippocampal gyrus (PhG, Fig 5D). Each of these hubs are in separate graph clusters, determined by the Louvain method.

For the AAL atlas (bottom section, Table 1), the three NPDR methods yield a consensus of ROIs selected by the LASSO method. These regions include the dorsal and ventral default mode networks (DMNs) and the right executive control network (ECN). The random forest centrality method includes multiple blocks of correlated variables (bottom, Table 1) in regions such as the anterior salience, auditory, and dorsal DMN. The NPDR methods have reduced multicolinearity compared to the random forest based method, and LASSO NPDR automatically selects a parsimonious set of ROIs.

The reduced multicollinearity in LASSO NPDR selected features can also be seen in the Brainnetome atlas (top, Table 1), where the LASSO NPDR rank list includes MTG_R_4_2 while non-LASSO NPDR and random forest also include the left hemisphere ROI, MTG_L_4_2. The variance inflation factor (VIF) for the correlation-based variable MTG_L_4_2–SPL_L_5_4 is greater than 5, suggesting the left MTG_4_2 ROI maybe involved in collinearity with other ROIs. Hemisphere symmetry in functional connectivity may lead to collinearity, and stronger connectivity in one hemisphere may lead to hemisphere-specific collinearity as in the case of MTG_L.

Analyses were performed using the Brainnetome (BNA) Atlas (top table) and the AAL atlas (bottom table). The feature selection methods used include correlation-diff NPDR with the LASSO penalty, correlation-diff-NPDR with the FDR adjusted P-value, centrality NPDR with adjusted P-value and degree, and centrality random forest with degree (top values chosen to match the length of the other methods). ROIs involving the MTG (middle temporal gyrus) are highlighted across methods (columns in top section).

## Discussion

The middle temporal gyrus (MTG) was found by all feature selection methods to be important for predicting MDD (Table 1). The centrality random forest method identified only the left MTG, while the LASSO correlation-diff NPDR method identified only the right MTG. The other NPDR methods, including the centrality-based method, identified both the left and right MTG. MTG is critical for semantic memory processing, visual perception, and language processing [23], and studies have shown associations with MDD. For example, studies using structural and functional MRI have identified significant gray matter abnormalities in the right MTG in participants with treatment-resistant depression (TRD) and treatment-responsive depression (TSD) compared to healthy controls [21]. The reduced gray matter volume in the bilateral MTG is indicative of structural changes associated with MDD. Similarly, a previous study showed that the fractional amplitude of low-frequency fluctuation (fALFF) in the right and left MTG was greater in participants with MDD than in HCs [22].

All of the tested methods showed that the superior temporal gyrus (STG) and inferior temporal gyrus (ITG) were important for predicting MDD. In participants with anxious depression versus healthy controls, a previous study found increased fALFF values in the left STG [24]. Although not previously linked to MDD, the ITG showed gray matter volume reductions in the MTG and ITG in chronic schizophrenia participants [25]. The link to another psychiatric condition could indicate a broader role for ITG in mood disorders.

## Conclusions

The application of machine learning and feature selection algorithms to fMRI data is increasingly critical for understanding the biological mechanisms of disorders. New methods are needed that can account for interactions between variables and regions of interest. We extended NPDR, with its ability to detect interactions, to handle data where the predictors are correlations between pairs of ROIs. We applied these NPDR centrality methods and a random forest centrality approach to correlation predictor data from a real rs-fMRI dataset for MDD, and the consensus between these methods found MTG, ITG, and STG to be important MDD ROIs.

We also developed a new simulation approach to compare correlation-based feature selection methods. We found that centrality NPDR scores with degree (i.e., Eq. 1 c-npdr with c = degree) are similar to scores based on correlation-diff NPDR (Eq. 2). This suggests a mathematical connection between the correlation-diff metric (Eq. 2) and degree centrality. The correlation-diff metric also might be improved by incorporating properties of other centralities. Degree centrality with NPDR gave better simulation performance than betweenness or eigenvector centrality. However, this difference could be due to the nature of the random networks used in the data simulation. Different network types, such as Watts-Storgatz small world networks, may simulate data where betweenness plays a more important. Future work will explore the effect network simulation parameters on feature selection performance.

## Abbreviations

rs-fMRI: resting-state functional magnetic resonance imaging
MDD: major depressive disorder
HC: healthy control
ROI: region of interest.
